# Organic Amendments Improved the Productivity and Bio-Fortification of Fine Rice by Improving Physiological Responses and Nutrient Homeostasis under Salinity Stress

**DOI:** 10.3390/plants12081644

**Published:** 2023-04-13

**Authors:** Imran Khan, Sikandar Mahmood, Muhammad Umer Chattha, Muhammad Bilal Chattha, Shahbaz Ahmad, Masood Iqbal Awan, Fatmah M. Alqahtani, Mohamed Hashem, Haifa Abdulaziz Sakit Alhaithloul, Sameer H. Qari, Faisal Mahmood, Muhammad Umair Hassan

**Affiliations:** 1 Department of Agronomy, University of Agriculture Faisalabad, Faisalabad 38040, Pakistan; 2 Department of Agronomy, Faculty of Agriculture Sciences, University of the Punjab, Lahore 54000, Pakistan; 3 Department of Entomology, Faculty of Agriculture Sciences, University of the Punjab, Lahore 54000, Pakistan; 4 Department of Agronomy, Sub-Campus Depalpur, University of Agriculture Faisalabad, Okara 38040, Pakistan; masood.awan@uaf.edu.pk; 5 Department of Biology, College of Science, King Khalid University, Abha 61413, Saudi Arabia; 6 Biology Department, College of Science, Jouf University, Al-Jouf 74331, Saudi Arabia; 7 Department of Biology, Al-Jumum University College, Umm Al-Qura University, Makkah 21955, Saudi Arabia; 8 Department of Environmental Sciences, Government College University Faisalabad, Faisalabad 38040, Pakistan; 9 Research Center on Ecological Sciences, Jiangxi Agricultural University, Nanchang 330045, China

**Keywords:** anti-oxidants, growth, hydrogen peroxide, press-mud, potassium influx, yield

## Abstract

Salinity stress (SS) is major abiotic stress that is seriously limiting crop production across the globe. The application of organic amendments (OA) mitigate the effects of salinity and improves soil health and crop production on a sustainable basis. However, limited studies are conducted to determine the impact of farmyard manure (FYM) and press mud (PM) on the performance of rice crop. Therefore, we performed this study to determine the impacts of FYM and PM on the growth, physiological and biochemical attributes, yield, and grain bio-fortification of rice crop under SS. The experiment was comprised of SS levels; control, 6 and 12 dS m^−1^ SS and OA; control, FYM: 5%, press mud 5% and combination of FYM (5%) + PM (5%). Soil salinity imposed deleterious impacts on the growth, yield, and grain quality of rice, however, OA appreciably offset the deleterious impacts of SS and improved the growth, yield, and grain bio-fortification of rice crop. The combined application of FYM + PM improved the growth and yield of rice through an increase in chlorophyll contents, leaf water contents, anti-oxidant activities (ascorbate peroxidise: APX; catalase: CAT, peroxidise: POD and ascorbic acid: AsA), K^+^ accumulation and decrease in Na^+^/K^+^ ratio, electrolyte leakage, malondialdehyde (MDA), hydrogen peroxide (H_2_O_2_), Na^+^ accumulation. Moreover, the combined application of FYM + PM significantly improved the grain protein (5.84% and 12.90%), grain iron (40.95% and 42.37%), and grain zinc contents (36.81% and 50.93%) at 6 and 12 dS m^−1^ SS. Therefore, this study suggested that the application of FYM and PM augmented the growth, yield, physiology, biochemistry, and grain bio-fortification of rice and proved to be a good practice for better rice production in salt-affected soils.

## 1. Introduction

Salinity stress is a potential abiotic stress that is significantly limiting crop growth and productivity across the globe [1]. Approximately 20% of soils across the globe are salt-affected and this percentage is projected to be increased by 30% by the end of 2050 [2]. The effects of salt stress are increasing due to lower rainfall and higher atmospheric temperature, which are major reasons for lower productivity globally [3,4]. Soil salinity induces various negative impacts in plants ranging from reduction in germination to seedling vigor and vegetative growth [5,6]. Salinity stress also disturbs the plant’s functioning and induces ROS production, which damage membranes, and proteins and causes a reduction in nutrient uptake and subsequent growth [5,7,8]. Moreover, SS also disturbs plant photosynthetic pathways, synthesis of proteins, the glycolysis pathway, and nitrogen fixation, and all these changes induce early senescence and cause a reduction in growth and productivity [5,9,10,11]. The excessive accumulation of salt in the rhizosphere disrupts osmotic pressure and ionic balance and alters the water uptake, transpiration, photosynthetic efficiency, and plant-relative water contents [12,13,14]. All these salt-induced changes cause a reduction in growth and productivity and even lead to plant death in case of higher salinity stress [15]. Therefore, there is a dire need to adopt appropriate practices to mitigate the adverse impacts of SS in order to ensure global food security.

The incorporation of organic amendments (OA) is considered an imperative practice to improve the soil properties and mitigate the deleterious impact of SS [16,17]. The positive effects of OA are associated with the addition of carbon, which allows microbial cells to counter the osmotic stress by adjusting the production of osmolytes under different stresses [18,19]. Farmyard manure (FYM) is an important OA and its field application markedly improved soil fertility and crop production [20,21]. The application of FYM improved the soil bulk density and soil organic matter contents and reduced soil electrical conductivity (EC) and sodium absorption ratio (SAR), resultantly improve growth and biomass production under salty conditions [22]. FYM also replaces Na^+^ in soil with Ca^2+^, which leads to a reduction in Na^+^-induced deleterious impacts on plants grown under SS [23]. FYM is used to increase the soil organic carbon contents [23,24] and an increase in soil organic carbon decreases the amount of Na^+^ in soil by absorption of Na^+^ with soil colloids and leading to a reduction in salt-induced negative impacts [25]. Press mud is also an important OA that is produced in large quantities as a by-product of the sugar industry. The application of PM improved the soil organic matter, nutrient cycling, microbial activities, and overall soil fertility and crop productivity [26,27,28,29]. The field application of PM improves nutrient uptake, plant water status, membrane integrity, plant photosynthetic efficiency, and osmolyte accumulation, resulting in a significant improvement in plant performance under SS [30,31]. Moreover, PM allows the better availability of Ca^2+^ in soil solution by mobilizing CaCO_3_, which in turn improves the soil structure stability, and increases Na^+^ leaching in salt-affected soils [32]. Thus, PM could be an important OA to improve crop productivity under SS.

Rice is an important crop globally and it is considered a global grain owing to the fact that it fulfills the food needs of more than three billion people [33,34,35,36,37]. Rice is a salt-sensitive cereal crop and SS seriously reduces growth as well as final productivity [38]. Salt stress negatively affects plant physiology and biochemistry in all developmental stages of rice starting from germination to senescence [39]. Salt stress also induces ionic and osmotic stress, which causes oxidative stress and nutrient depletion [40,41]. However, cell signaling in rice plants allows for an increase in Na^+^ exclusion, osmolyte accumulation, and antioxidant activities that increase salt tolerance in rice [42,43].

However, the role of FYM, PM, and the combination of FYM and PM to alleviate the deleterious impacts of SS is not well explored. Therefore, to fill this gap of knowledge we hypothesized that FYM in combination with PM can reduce the toxic impacts of SS on rice crops by improving plant physiological and biochemical responses and nutrient homeostasis. Consequently, the present study was performed to determine the impact of FYM and PM on growth, physiological traits, anti-oxidant activities, yield, and grain bio-fortification of rice grown in saline soil.

## 2. Materials and Methods

### 2.1. Experimental Details

The present study was performed in pots at the wire house of the University of Agriculture, Faisalabad, Pakistan. The study site has semiarid conditions with a mean maximum temperature of 44.3 °C and a mean minimum temperature of 32.2 °C, rainfall of 40 mm, and relative humidity of 42.3%. The study was conducted in a CRD factorial arrangement having four replications and 48 experimental units. The study consisted of diverse SS levels; control, 6 and 12 dS m^−1^ SS, and different OA; control, 5% FYM, 5% PM, and FYM (5%) + PM (5%). The soil was collected from the agronomy farm (31.8° N, 73.8° E, 184 m asl) and sieved to remove any debris. The soil was clay loam with pH 7.84, EC 0.92 dS m^−1^, organic matter 8.12 g kg^−1^, total nitrogen (N) 0.32 g kg^−1^, and available phosphorus (P) and potassium (K) 9.52 and 162 mg kg^−1^. After that, pots were filled with soil and silt having a 2:1 ratio. The twenty-eight-day-old nursery of rice variety (Kissan Basmati) was collected from a farmer’s field and grown in plastic pots with dimensions of 36 cm × 33 cm and capacity of 8 kg during the 3rd week of June. Five rice seedlings were transplanted into each pot and were regularly watered. For achieving the desired levels of 5% FYM and 5% PM; we added 500 g of each FYM and PM in each pot 7 days before translating the seedlings. PM used in the study was taken from Madina Sugar Mills Limited, Faisalabad it has 304.2 g kg^−1^ carbon on a dry weight basis (DW), N 105 g kg^−1^ DW, P 70 g kg^−1^ DW, K 50 g kg^−1^ DW, and had pH 6.84. FYM manure was taken from the agronomy field and it contained carbon 101 g kg^−1^ DW, N 68.5 g kg^−1^ DW, P 59.2 g kg^−1^ DW, potassium 43.2 g kg^−1^ DW, and it had a pH of 6.39.

To achieve the desired SS levels, sodium chloride (*NaCl*) was used and the concentration of *NaCl* was determined according to treatments by procedures of Khan et al. [19].

$$NaCl\;required\;\left(g/kg\right)\frac{TSS\times 58.5\times saturation\;\left(\%\right)}{100\times 100}$$

TSS indicates the total soluble salts and it was measured by multiplying the EC difference (required soil EC−initial soil EC) with a factor of 10 and *NaCl* molecular weight. The amount of *NaCl* required to develop 6 dS m^−1^ and 12 dS m^−1^ salinity levels was 9.4 g/8 kg and 18.9 g/8 kg of pot respectively. For determination of soil saturation; 250 g soil was taken and paste was made and kept at room condition for 2 h. Afterward, the extract was obtained and soil saturation was determined with the following formula:

$$saturation\;\left(\%\right)\frac{loss\;in\;soil\;weight\;on\;drying}{weight\;of\;soil\;after\;drying}\times 100$$

### 2.2. Measurement of Morphological Traits

For the determination of morphological parameters, three plants randomly from each treatment were taken and shoot length (SL) was measured from base to flag leaf tip with the help of measuring tape. Moreover, the length of roots was measured with measuring tape, and leaves and tillers per plant were counted manually. The plants were finally collected and measured the fresh and dry weight of root and shoot.

### 2.3. Determination of Relative Water Contents, Electrolyte Leakage, and Photosynthetic Pigments

For the determination of plant physiological and biochemical parameters plant samples were taken at the flag leaf stage (54 days after nursery transplanting). The top leaves from plants were taken and weighed to count the fresh weight (FW), then leaves were dipped in water for 24 h to take the turgid weight (TW). Later on, these leaves were oven dried (80 °C) and dry weight (DW) was taken and relative water content (RWC) was determined as follows: RWC = (FW − DW)/(TW − DW) × 100. To measure EC, 0.5 g of rice leaf samples were soaked in water for a time period of 24 h and EC1 was measured with an EC meter (Model DJS-1C Model DJS-1C; Shanghai Analytical Instrument Co. Shanghai, China) then leaves were auto-calved (120 min) and allowed to reach equilibrium and EC2 was measured and EL% was calculated with following equation: EL%=(EC1÷EC2 )×100.

0.5 g rice plant samples were taken and chopped into pieces and dipped in 80 mL acetone solution for 24 h at room temperature to settle down. After 24 h, chlorophyll a and b and carotenoid contents were measured through a spectrophotometer at 663 nm, 645 nm, and 480 nm wavelengths respectively [44].

### 2.4. Determination of Potential Osmolytes, Oxidative Stress Markers and Antioxidant Activities

0.5 g rice leaf samples were ground in phosphate buffer (5 mL) and centrifuged at 14,000 rpm for 15 min to collect the supernatant. Later, plant samples were treated with 2 mL Bradford reagent and absorbance was noted by a spectrophotometer at 595 nm to determine the total soluble proteins (TSP) [45]. To measure the free amino acids (FAA), fresh rice samples (0.5 g) were extracted with phosphate buffer (0.2 M). After that, the extract was added in 1 mL of each pyridine and ninhydrin, and absorbance was taken at 570 nm for the determination of FAA [46].

Rice fresh leaf samples (0.5 g) were taken and ground with 0 5 mL of trichloroacetic acid (TCA) and the supernatant was obtained to determine hydrogen peroxide (H_2_O_2_) concentration. Afterward, 1 M of potassium iodide (KI) and potassium phosphate buffer (1 mL) was added to the supernatant of the crude extract and kept for 30 min and absorbance noted at 390 nm [47]. In the case of malondialdehyde (MDA) determination, the leaf sample was homogenized using 5 mL TCA and kept for 15 min at 12,000 RPM. The supernatant was added in 5 mL of thiobarbituric acid (TBA) and boiled for 30 min at 100 °C and then cooled quickly and absorbance was noted at 532 and 600 nm. To determine the antioxidant activities plant samples were taken at the flag leaf stage. Then, 0.5 g fresh leaves was dipped in a test solution carrying 5 mL potassium phosphate buffer and 5.9 mM H_2_O_2_ for 24 h. After 24 h, the sample was extracted at 10,000 rpm and the absorbance was noted at 240 nm to determine catalase (CAT) contents [48]. For peroxidase (POD) determination, 0.5 g fresh leaves were ground in potassium phosphate buffer (0.5 mM) and centrifuged at 15,000 rpm for 15 min and the supernatant was taken and 0.5 mM ascorbic acid (100 μL) and 6.5 mM of H_2_O_2_ (100 μL) were added. The absorbance was measured through a spectrophotometer at 290 nm to determine POD activity [49]. To measure ascorbate peroxidases (APX) 0.5 g of fresh leaves were homogenized in 5 mL potassium phosphate buffer with pH 7.8 and centrifuged for 5 min and absorbance was taken at 470 nm. For the determination of ascorbic acid, 0.5 g leaf was homogenized by using 10% TCA (5 mL) at 8000 RPM for ten minutes. After incubation, 2 mL of H_2_SO_4_ was added to the solution and kept for 30 min and the absorbance was noted at 520 nm [50].

### 2.5. Determination of Yield and Quality Traits

The number of panicles bearing tillers was counted randomly from four replications of each treatment. Likewise, panicles of all plants in each pot were taken and their lengths were measured. The number of grains of each panicle of each pot was counted and the grains of each pot were collected and weighed separately. The rice grains were oven-dried and ground to determine the protein contents. The ground grain samples were sieved (0.5 mm) and digested by adding a mixture of acids (2:1, HNO_3_/HClO_4_). The concentration of iron and zinc was measured using an atomic absorption spectrophotometer (Hitachi U-2001, Tokyo, Japan).

### 2.6. Statistical Analysis

The data on all the collected traits were subjected to two-way ANOVA for SS, OA, and their interaction. The data were analyzed using the analysis of variance and least significant difference test (LSD) was used to compare the mean values of different treatments [51]. Data were graphically plotted by using Sigma plot software.

## 3. Results

### 3.1. Organic Amendments Improve Rice Growth under SS

The results showed that SS and OA had a significant impact on the growth traits of rice (Table 1). Salinity stress significantly reduced the root and shoot growth of rice plants (Table 1). RL and SL were reduced by 27.6% and 54.21% at 12 dS m^−1^, similarly, leaves/plant, tillers, SFW, SDW, RFW, and RDW were also reduced by 60.6%, 53.3%, 69.36%, 53.35%, 61.37% and 60.90% 12 dS m^−1^ (Table 1). However, the application of PM and FYM improved all the growth traits under saline conditions (Table 1). The combined use of FYM and PM improved the shoot and root length by 10% and 25%, leaves/plant, tillers by 20%, SFW and RFW by 65% and 30% and RDW and SDW by 27% and 44%, respectively, at 12 dS m^−1^ SS compared with control (Table 1). SS (12 dS m^−1^) significantly reduced the root and shoot growth of rice, however, FYM + PM offset the toxic effects of salinity and improved root and shoot growth.

### 3.2. Organic Amendments Improve Photosynthetic Pigments and RWC and Reduce EL under SS

**Figure 1:**
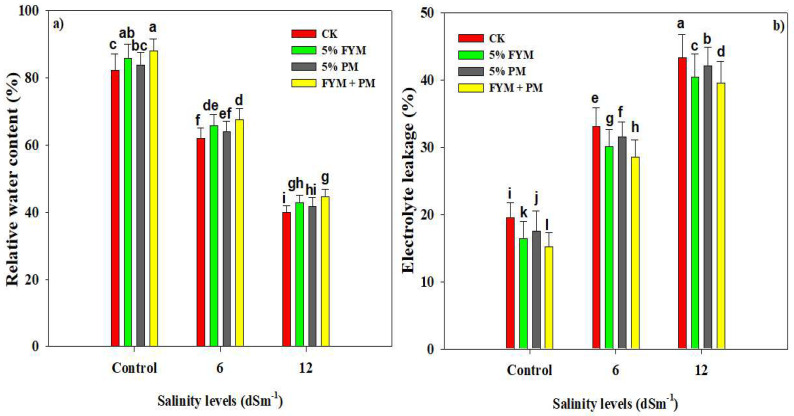
Effect of different organic amendments on relative water contents (**a**) and electrolyte leakage (**b**) under diverse leaves of salinity stress. The bars are means of four replicates with ±SE while diverse letters indicate significant differences at *p* < 0.05.

Elevating levels of SS significantly decreased the photosynthetic pigments in rice plants (Table 2). A reduction of 18.6%, 35.3%, and 61.9% in chlorophyll a, chlorophyll b, and carotenoid contents was observed under 12 dS m^−1^ (Table 2). On the other hand, PM (5%) and FYM (5%) significantly increased the aforementioned photosynthetic pigments as compared with the control. An increase of 32%, 8.31%, 21.14%, and 19.14% in chlorophyll a, b, total chlorophyll, and carotenoid concentration was recorded with combined FYM (5%) and PM (5%) under 12 dS m^−1^ as compared with control (Table 2). RWC markedly decreased under SS conversely while a significant increase in the EL was recorded under different levels of SS (Figure 1). RWC contents were decreased by 58.5% at 12 dS m^−1^ SS while EL was increased by 50.2% at 12 dS m^−1^ SS compared with the control (Figure 1). However, the combined application of FYM (5%) and PM (5%) decreased the EL by 8.02% and increased the RWC by 13.3% under 12 dS m^−1^ SS (Figure 1). Salinity stress caused a substantial reduction in photosynthetic pigments and RWC and increased the EL, however, FYM + PM appreciably improved the synthesis of photosynthetic pigments, RWC, and reduced EL.

### 3.3. Organic Amendments Maintain a Lower H_2_O_2_ and MDA Accumulation and Increase Accumulation of TSP and FAA in Rice under SS

**Figure 2:**
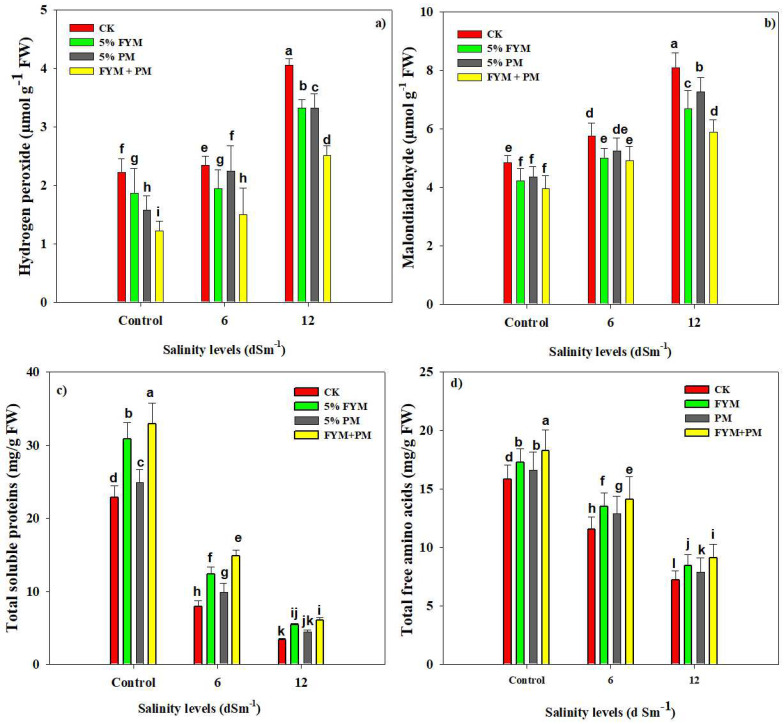
Effect of different organic amendments on H_2_O_2_ (**a**) MDA (**b**), TSP (**c**), and FAA (**d**) under diverse leaves of salinity stress. The bars are means of four replicates with ±SE while diverse letters indicate significant differences at *p* < 0.05.

H_2_O_2_ production was significantly elevated with increasing levels of SS (Table 2). The maximum concentration of H_2_O_2_ (4.08 µmol) was noted at stronger SS (12 dS m^−1^) without FYM + PM application and minimum H_2_O_2_ (1.22 µmol) was recorded in control. The application of FYM (5%) + PM (5%) reduced the H_2_O_2_ content by 39.5% at the 12 dS m^−1^ SS compared to the control (Figure 2).

Salt stress also caused a significant increase in MDA accumulation and this increase was linearly increased with increasing the concentration of salt stress in the growth medium (Figure 2). The application of FMY (5%) + PM (5%) significantly reduced MDA accumulation as compared to the control (Figure 2). Salinity stress exhibited a substantial reduction in the TSP and FAA (Figure 2). A sharp reduction in TSP and FAA by 83.5% and 52.0% was recorded at 12 dS m^−1^ as compared to the control (Figure 2). However, FYM + PM boosted the TSP and FAA concentration under SS and control conditions (Figure 2). To sum up, SS increased the MDA and H_2_O_2_ production and reduced TSP and FAA, while FYM + PM (5%) mitigated MDA and H_2_O_2_ production and increased TSP and FAA which reduced the negative impacts of SS.

### 3.4. Organic Amendments Improve Antioxidant Activities in Rice under SS

The results revealed that SS increased antioxidant activities as compared to the control treatment (Table 3), similarly, FYM + PM also significantly increased the antioxidant activities under SS (Table 3). The activities of CAT and APX were increased by 59.9% and 68.8% respectively, at 12 dS m^−1^ as compared to control, while activities of POD and AsA increased by 15.9% and 37.4% under 12 dS m^−1^ SS, respectively, as compared with control (Table 3). The application of FYM (5%) + PM (5%) significantly increased APX, CAT, POD, and AsA activity by 89.53%, 20.40%, 66.66%, and 14.88%, respectively, under 12 ds m^−1^ SS as compared with control (Table 3 The combined use of FYM (5%) + PM (5%) proved more beneficial in increasing antioxidant activities).

### 3.5. Organic Amendments Maintain Ionic Homeostasis under SS

**Figure 3:**
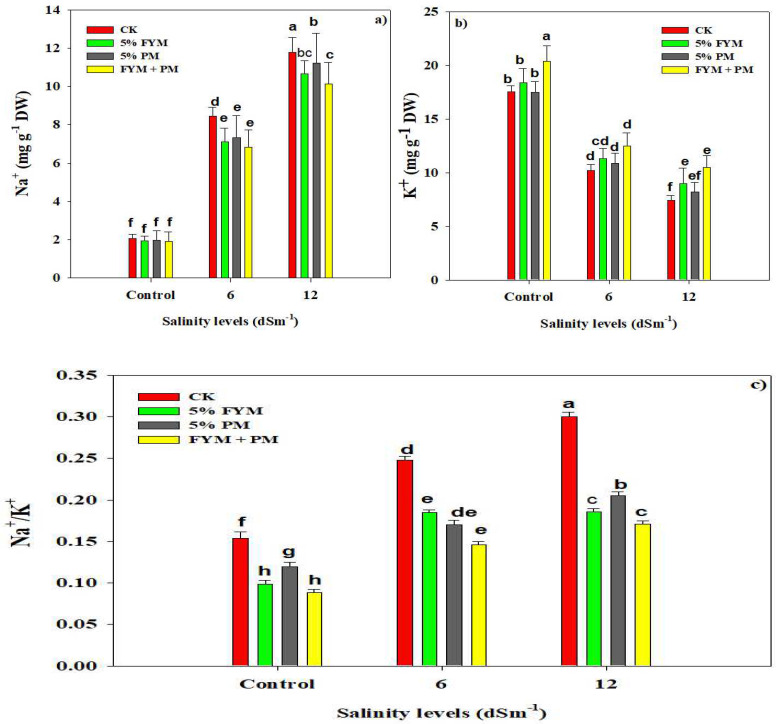
Effect of different organic amendments on Na^+^ (**a**) and K^+^ concentration (**b**) and Na^+^/K^+^ (**c**) under diverse leaves of salinity stress. The bars are means of four replicates with ±SE while diverse letters indicate significant differences at *p* < 0.05.

Salinity stress significantly increased Na^+^ accumulation and reduced K^+^ accumulation (Table 4). Na^+^ accumulation was increased by 277.16% and 455.83% under 6 and 12 dS m^−1^ SS while K^+^ accumulation was decreased by 64.52% and 110% under 6 and 12 dS m^−1^ SS (Figure 3). The application of OA maintained a higher K^+^ accumulation and appreciably reduced Na^+^ accretion in rice plants (Figure 3). The combined application of FYM (5%) and PM (5%) significantly enhanced K^+^ accumulation and decreased the Na^+^ accumulation under both levels of SS (Figure 3). SS also increased the Na^+^/K^+^ ratio; however, the application of FYM (5%) + PM (5%) significantly reduced the Na^+^/K^+^ ratio as compared with FYM alone, PM application alone, and control (Figure 3). Thus, the combined use of FYM (5%) + PM (5%) maintained a better K^+^ uptake and reduced the Na^+^ accumulation and Na^+^/K^+^ ratio, which improved the rice growth (Figure 3).

### 3.6. Organic Amendments Increased the Yield of Rice under SS

SS noticeably reduced the yield and yield parameters of rice crops (Table 4). The panicle length, panicles plant^−1^, grains panicle^−1^, and grain yield were decreased by 41.63%, 70.28%, 35.18%, and 87.8%, respectively at 12 dS m^−1^ SS as compared with control (Table 4). OA significantly improved yield traits of rice crop (Table 4); an increase of 15.76% and 36.01% in panicle length and panicles plant^−1^, respectively, was found with combined FYM (5%) and PM (5%) at 12 dS m^−1^ SS compared with control. Likewise, grains panicle^−1^ and grain yield pot^−1^ also increased by 9.83% and 15.58%, respectively, with combined FYM and PM applications as compared with control (Table 4).

### 3.7. Organic Amendments Increase Grain Bio-Fortification under SS

Salinity stress considerably reduced the grain protein, zinc (Zn), and iron (Fe) contents (Table 5). A maximum reduction of 13.10% and 21.78 in grain protein was recorded at 6 and 12 dS m^−1^ SS respectively (Table 5). However, the application of FYM (5%) and PM (5%) significantly increased the grain protein contents by 5.84% and 12.90% at moderate and stronger SS (Table 5). Likewise, grain Fe and Zn contents also decreased by 15.10% and 30.29% at 6 dS m^−1^ SS and 11.84% and 18.82% under 12 dS m^−1^ SS (Table 5). The combined application of FYM (5%) and PM (5%) significantly increased the grain Fe (40.95% and 42.37%) and Zn (36.81% and 50.93%) under 6 and 12 dS m^−1^ SS, respectively as compared with control (Table 5). The combined use of FYM + PM effectively improved the grain protein, and grain Fe and Zn concentration under saline conditions as compared to the control and alone application of FYM and PM alone.

## 4. Discussion

Salt stress is a major limiting factor for crop production across the globe; therefore, it is essential to improve salt tolerance in plants for ensuring better food production. The results indicated that SS induced a significant reduction in the growth and biomass productivity of rice crop (Table 1). Salt stress disrupted nutritional balance (Figure 3) and induced the production of ROS (Figure 2) which leads to a substantial reduction in growth and biomass production [52,53]. Salt stress induced a significant increase in Na^+^ and Cl^−^ accumulation around the plant roots, which reduced the uptake of water owing to an increase in the osmotic pressure of soil solution and resultantly reduced plant growth [54]. The application of different OA significantly reduced the impacts of SS and caused a prominent increase in rice growth (Table 1). The application of OA improved SOM and led to an appreciable increase in soil water hold capacity, hydraulic conductivity, soil bulk density, and nutrient availability [55], thereby improve plant growth and productivity under SS (Table 1). The addition of OM following OA application improved the Na^+^ leaching in soil, which reduce Na^+^-induced oxidative damages, therefore improving plant growth and biomass production [56,57]. The current findings are the same as the results of [19]; they also noted that PM improved rice growth and biomass under SS. The chlorophyll contents of rice plants were significantly decreased under SS (Table 2). SS enhances chlorophyll-degrading enzyme (chlorophyllase) activity, thereby reduce chlorophyll content.

The reduction in chlorophyll content owing to SS has been reported in many crops including maize, sunflower, and rice [58,59]. Additionally, SS induces oxidative stress in chloroplasts and reduces chlorophyll content due to the de-naturation of enzymes responsible for the synthesis of chlorophyll content [58,60]. In the current study, SS significantly reduced RWC (Figure 1) owing to salinity-induced osmotic stress which causes a reduction in water uptake. However, rice plants also have an ABA-independent DST-mediated pathway which enhances salt tolerance by reducing the stomata’s movement and density and improving the subsequent physiological processing [61]. Likewise, the application of diverse OA further improved the physiological functioning of rice plants under control and SS conditions (Table 2). The application of OA decreased the MDA and H_2_O_2_ accumulation and increased the Mg uptake, which in turn, improved the chlorophyll contents owing to the fact Mg plays a crucial role in chlorophyll synthesis [62,63,64]. We found that the application of OA also improved the RWC contents under both control and SS (Figure 1). This indicates that OA improved the soil OM contents and crafted favorable soil which increases the water uptake and leads to a significant increase in RWC under SS [31,65].

Salt stress significantly increased the concentration of oxidative stress markers in rice plants (Figure 2); however, the accumulation of these oxidative indicators was significantly increased with an increase in salt concentration (Figure 2). Previous reports showed that SS causes damage to the cell membrane [66,67] and an increase in membrane damage also causes a significant increase in EL. The excessive Na^+^ concentration favors ROS production (Figure 3) by working as a signaling molecule in the signal transduction pathway [58]. The rice plants respond to SS by perceiving ionic and osmotic signaling that allows them to withstand saline conditions [40,61,68]. The rice plants also regulate the salt-responsive genes and increase the biosynthesis of osmolytes and maintain Na^+^ efflux to counter the toxic effects of salt stress [69,70]. The study findings also indicated that the application of OA maintained the better biosynthesis of osmolytes and maintained lower Na^+^ concentrations that improved the salt tolerance in rice plants. Likewise, the application of OA caused a marked reduction in MDA and H_2_O_2_ accumulation (Figure 3) owing to improved anti-oxidant activities (Figure 3) and reduced Na^+^ uptake, Na^+^/K^+^ ratio, and increased K^+^ uptake [31]. Salt stress reduced TSS and FAA concentration (Figure 2); however, the application of OA caused a marked increase in both TSP and FAA under both salt and control conditions (Figure 2). These findings give evidence that OA increased the uptake of nitrogen which increased TSP owing to the fact N plays an imperative role in protein synthesis [19].

The increase in TSP regulates plant metabolic functioning and anti-oxidant activities which increased salt tolerance [11,71]. Moreover, OA-mediated increase in FAA accumulation creates an osmotic potential gradient that favors the inward water movement and reduces the toxicity of SS [11]. The results indicated that the activities of anti-oxidants were significantly increased under SS. Moreover, OA also caused a clear improvement in anti-oxidant activities under SS (Table 3). Indeed, an increase in antioxidant activities was observed under SS which indicates that plants under SS activate their defense system to cope with SS. Various scientists reported a substantial increase in anti-oxidant (APX, CAT, and SOD) activities and an increase in gene expression of these anti-oxidant enzymes in maize, pearl millet, and rice under SS [60,72,73]. The application of various OA improved the anti-oxidant activities; however, the application of FYM and PM remained the top performer in this context (Table 3). The favorable soil conditions and the addition of OM following OA might be the reason for this increase in anti-oxidant activities under SS. However, further study is needed to explore the mechanism lying behind the increase in anti-oxidant activities following the addition of OA.

For normal enzymatic functioning in plant cells, higher K^+^ and lower Na^+^ ions are essential in the cell cytoplasm [58]. SS increased Na^+^ concentration which decreased the K^+^ accumulation in rice leaves (Figure 3). The increase in Na^+^ is a major reason for the ionic balance owing to the fact that Na^+^ is a main toxic ion under SS [58,74]. The increase in salinity stress increased the Na^+^ accumulation, which inhibits the K^+^ uptake and accumulation in plants. Because of lower external proton concentration, SS reduced the capacity of Na^+^/K^+^ anti-porters to exclude Na^+^, thereby resulting in a higher accumulation of Na^+^ in plant cells [75,76,77]. However, the application of OA caused a marked reduction in Na^+^ accumulation while increased the K^+^ accumulation (Figure 3).

The addition of OM following OA application decreases Na^+^ ions owing to the fact that OM stores Na^+^ in the form of Na-organic compounds [78]. The reduction in Na^+^ ions favors an increase in K^+^ accumulation under SS. Na^+^/K^+^ is considered a good index for assessing the damages of SS. We noted that SS increased Na^+^/K^+^ which is consistent with the results of Xu et al. [79] and Khan et al. [19]; they also observed a significant increase in Na^+^/K^+^ under SS. However, OA caused a reduction in the Na^+^/K^+^ ratio, which indicates OA amendments decrease Na^+^ accumulation and increase K^+^ uptake, which maintains a lower Na^+^/K^+^ ratio [80,81].

SS markedly reduced the yield traits of rice crop (Table 4). The increased Na^+^ accumulation (Figure 3) due to SS induces early senescence and reduces assimilates production and panicle formation which causes a significant yield losses [82]. Moreover, SS disturbs photosynthesis, causes ionic imbalance and ROS production, and reduces nutrient uptake, RWC, and membrane integrity. Therefore, all these changes cause a marked reduction in crop yield [19,83]. However, the application of OA significantly increases the yield of rice plants (Table 4). Our study suggests that the application of OA improved soil properties, photosynthetic efficiency, nutrient uptake and anti-oxidant activities, K^+^ uptake, and lowered Na^+^/K^+^ which enhanced yield and yield traits of maize, wheat, and rice [64,84,85]. The results indicated that SS decreased the grain protein contents (Table 5). High Na^+^ accumulation interferes with nitrogen absorption resultantly reduced grain protein contents [86,87]. Zinc (Zn) and iron (Fe) are essential nutrients needed for humans and plants [27,88,89]. In the present study, SS caused a significant reduction in grain Fe and Zn contents (Table 5). The restricted Zn uptake limits rice growth and deteriorates grain quality [90,91]. Na^+^ and Cl^–^ ions in plant tissue compete with Zn^2+^ and reduce Zn uptake by plants, thereby causing a reduction in grain Zn concentration [92]. However, OA significantly improved the grain protein and grain Fe and Zn contents (Table 5). OA improves soil OM, which provides a substrate for decomposing microbes and resulted in better nutrient uptake and their subsequent accumulation in grains in rice plants [65,93].

## 5. Conclusions

Soil salinity reduced the growth, yield, and quality of rice through an increase in H_2_O_2_, MDA, and Na^+^ accumulation and reduced photosynthetic pigments, K^+^ uptake, and leaf water contents. The combined use of FYM and PM offset the negative effects of salinity and improved the yield and quality through an increase in photosynthetic pigments, K^+^ uptake, leaf water contents, antioxidant activities, and reductions in H_2_O_2_, MDA and Na^+^ accumulation and the Na^+^/K^+^ ratio. The present findings indicate that FYM and PM could be effective strategy to ameliorate salinity stress for rice crops. However, more field studies are needed under diverse climatic and soil conditions before its use on a large scale.

## Figures and Tables

**Table 1:** Effect of different organic amendments on the rice growth traits under diverse salt stress levels.

Salinity Stress	OA	PH (cm)	RL (cm)	SFW (g)	RFW (g)	SDW (g)	RDW (g)	LPP
Control	Control	76.91d ± 0.21	7.60c ± 0.21	23.75c ± 0.25	4.08d ± 1.20	13.92bc ± 0.52	2.25de ± 0.40	28.58d ± 0.34
	5% FYM	82.17b ± 0.22	9.33ab ± 0.70	24.58a ± 0.16	5.29b ± 0.44	15.00b ± 0.76	4.00b ± 0.36	32.67b ± 0.82
	5% PM	79.17c ± 0.09	8.75b ± 0.25	24.00b ± 0.59	4.71c ± 0.56	14.00c ± 0.24	3.00c ± 0.14	30.42c ± 0.57
	FYM + PM	85.19a ± 0.41	10.07c ± 0.38	25.33a ± 0.24	5.95a ± 0.62	16.92a ± 0.88	4.83a ± 0.29	35.33a ± 0.65
6 dS/m	Control	64.00h ± 0.36	5.35g ± 0.06	13.16de ± 0.22	2.62gh ± 0.50	9.50ef ± 0.65	1.42f ± 0.08	19.75g ± 0.33
	5% FYM	69.16f ± 0.32	6.63de ± 0.26	17.25cd ± 0.29	3.25ef ± 0.37	11.00de ± 0.49	2.45cd ± 0.14	22.50g ± 0.22
	5% PM	66.25g ± 0.25	5.96ef ± 0.45	15.08de ± 1.05	2.96fg ± 0.36	10.41e ± 0.46	2.12de ± 0.12	21.00g ± 0.72
	FYM + PM	72.00e ± 0.47	7.35cd ± 0.14	19.00c ± 0.76	3.70de ± 0.40	12.58cd ± 0.32	2.75cd ± 0.16	24.00e ± 0.59
12 dS/m	Control	55.50j ± 0.62	3.54i ± 0.19	6.16h ± 0.35	1.62j ± 0.35	4.25h ± 0.69	1.31f ± 0.04	10.50j ± 0.29
	5% FYM	58.91i ± 0.42	4.25hi ± 0.19	7.92fg ± 0.28	1.99ij ± 0.61	8.42g ± 0.25	1.32f ± 0.23	13.16i ± 0.35
	5% PM	57.58ij ± 0.71	3.79i ± 0.16	7.08gh ± 0.58	1.87ij ± 0.22	6.75g ± 0.55	1.26f ± 0.11	11.87i ± 0.19
	FYM + PM	62.08h ± 0.55	4.79gh ± 0.42	8.75ef ± 0.38	2.26hi ± 0.26	8.50f ± 0.56	1.62ef ± 0.21	14.50h ± 0.22

PH: plant height, RL: root length, SFW: shoot fresh weight, RFW: root fresh weight, RFW: root fresh weight, RDW: root dry weight, LPP: leaves per plant, OA: organic amendments, FYM: farmyard manure, PM: press mud. Values presented in the tables are the mean of four replicates with ±SE and different letters with each value indicating a significant difference at *p* < 0.05.

**Table 2:** Effect of different organic amendments on photosynthetic pigments under diverse salt stress levels.

Salinity Stress	OA	Chlorophyll a (mg/g FW)	Chlorophyll b (mg/g FW)	Carotenoids (mg/g FW)
Control	Control	0.54c ± 0.0011	0.33b ± 0.0063	3.70d ± 0.072
	5% FYM	0.57b ± 0.0018	0.35a ± 0.0027	4.11b ± 0.028
	5% PM	0.55c ± 0.0014	0.34b ± 0.0030	3.86c ± 0.086
	FYM + PM	0.59a ± 0.0030	0.36a ± 0.0054	4.34a ± 0.074
6 dS/m	Control	0.44d ± 0.0032	0.27cd ± 0.0067	2.48g ± 0.055
	5% FYM	0.46d ± 0.0018	0.28c ± 0.0054	2.86f ± 0.072
	5% PM	0.45d ± 0.0016	0.28c ± 0.0039	2.73f ± 0.086
	FYM + PM	0.47d ± 0.0011	0.30c ± 0.0049	3.06e ± 0.033
12 dS/m	Control	0.38f ± 0.0027	0.21f ± 0.0066	1.18k ± 0.074
	5% FYM	0.39f ± 0.0014	0.23e ± 0.0047	1.61i ± 0.049
	5% PM	0.38f ± 0.0024	0.22e ± 0.0029	1.35j ± 0.037
	FYM + PM	0.41e ± 0.0012	0.23e ± 0.0064	1.94h ± 0.034

OA: organic amendments, FYM: farmyard manure, PM: press mud. Values presented in the tables are the mean of four replicates with ± SE and different letters with each value indicating a significant difference at *p* < 0.05.

**Table 3:** Effect of different organic amendments on anti-oxidant activities under diverse salt stress levels.

Salinity Stress	OA	APX (U/mg Protein)	CAT (U/mg Protein)	POD) (U/µg Protein)	Ascorbic Acid (mg/g FW)
Control	Control	6.62g ± 0.40	1.83l ± 0.30	0.13j ± 0.0052	15.14l ± 0.47
	5% FYM	6.90g ± 0.67	2.24k ± 0.12	0.18h ± 0.0006	17.74j ± 0.20
	5% PM	7.13g ± 0.21	2.74j ± 0.087	0.15i ± 0.0026	16.41k ± 0.27
	FYM + PM	7.40g ± 0.16	3.21i ± 0.082	0.20g ± 0.0034	19.07i ± 0.14
6 dS/m	Control	19.96f ± 0.22	3.68h ± 0.14	0.25f ± 0.0010	20.34h ± 0.19
	5% FYM	21.31f ± 0.37	4.15g ± 0.012	0.30d ± 0.0030	22.93f ± 0.11
	5% PM	21.87f ± 1.57	4.62f ± 0.16	0.27e ± 0.0020	21.54g ± 0.23
	FYM + PM	26.83e ± 0.75	5.09e ± 0.21	0.38b ± 0.0015	24.20e ± 0.24
12 dS/m	Control	20.44d ± 0.51	5.55d ± 0.22	0.27e ± 0.0023	25.59d ± 0.40
	5% FYM	26.84c ± 0.48	6.02c ± 0.15	0.33c ± 0.0026	28.10b ± 0.40
	5% PM	32.71b ± 0.98	6.49b ± 0.13	0.25f ± 0.0012	26.86c ± 0.54
	FYM + PM	38.74a ± 1.32	6.96a ± 0.15	0.45a ± 0.0008	29.40a ± 0.34

OA: organic amendments, FYM: farmyard manure, PM: press mud. Values presented in the tables are the mean of four replicates with ±SE and different letters with each value indicating a significant difference at *p* < 0.05.

**Table 4:** Effect of different organic amendments on yield traits under diverse salt stress levels.

Salinity Stress	OA	TPP	GPP	PP	PL (cm)	GYPP (g)
Control	Control	5.75c ± 0.16	128.00d ± 0.45	11.00d ± 0.23	21.00cd ± 0.89	30.50d ± 0.44
	5% FYM	8.00b ± 0.24	132.00b ± 0.57	14.00b ± 0.49	23.08ab ± 0.21	33.17b ± 0.44
	5% PM	6.25c ± 0.08	130.00c ± 0.23	12.00c ± 0.36	22.16bc ± 0.32	31.92c ± 0.29
	FYM + PM	9.50a ± 0.21	135.00a ± 0.14	16.00a ± 0.36	24.25a ± 0.29	34.33a ± 0.52
6 dS/m	Control	4.08d ± 0.16	115.00g ± 0.56	6.00g ± 0.83	16.00gh ± 0.015	9.33h ± 0.57
	5% FYM	5.75d ± 0.25	120.00e ± 0.49	8.58e ± 0.167	18.20ef ± 0.42	11.50f ± 0.55
	5% PM	4.66d ± 0.49	118.00f ± 0.36	7.16f ± 0.417	17.17fg ± 0.29	10.42g ± 0.40
	FYM + PM	6.25c ± 0.41	122.00e ± 0.27	10.08d ± 0.360	19.37de ± 0.55	12.50e ± 0.24
12 dS/m	Control	2.25e ± 0.25	81.00i ± 0.24	3.00i ± 0.315	12.00k ± 0.53	3.50k ± 0.30
	5% FYM	4.00d ± 0.33	85.00gh ± 0.43	4.08h ± 0.518	13.58ij ± 0.33	4.00j ± 0.29
	5% PM	3.00e ± 0.40	83.00hi ± 0.24	3.50hi ± 0.215	12.70jk ± 0.34	3.85j ± 0.15
	FYM + PM	4.50d ± 0.21	88.00gh ± 0.27	5.16g ± 0.222	14.54hi ± 0.41	4.50i ± 0.21

TPP: tillers per plant, GPP: grains per panicle, PP: panicles per plant, PL: panicle length, GYPP: grain yield per pot. OA: organic amendments, FYM: farmyard manure, PM: press mud. Values presented in the tables are the mean of four replicates with ±SE and different letters with each value indicating a significant difference at *p* < 0.05.

**Table 5:** Effect of different organic amendments on grain quality traits under diverse salt stress levels.

Salinity Stress	OA	Protein (%)	Iron (mg/kg DW)	Zinc (mg/kg DW)
0	Control	6.65c ± 0.70	36.55e ± 1.54	21.20de ± 0.14
	5% FYM	6.93b ± 1.49	47.45b ± 1.68	26.20b ± 0.027
	5% PM	6.60c ± 1.21	42.40c ± 1.32	23.03c ± 0.074
	FYM + PM	7.11a ± 0.70	54.10a ± 1.17	29.70a ± 0.065
6 dS/m	Control	5.82e ± 0.47	32.30f ± 1.64	19.18f ± 0.074
	5% FYM	6.16d ± 0.47	41.35c ± 1.83	23.08c ± 0.066
	5% PM	5.99de ± 1.25	37.65de ± 0.93	21.03de ± 0.099
	FYM + PM	6.16d ± 2.16	45.53b ± 0.92	26.23b ± 0.027
12 dS/m	Control	5.27f ± 0.62	28.13g ± 0.77	16.53g ± 0.071
	5% FYM	5.81e ± 0.83	37.25e ± 1.36	22.40cd ± 0.067
	5% PM	5.37f ± 0.85	33.10f ± 0.84	20.38ef ± 0.072
	FYM + PM	5.95e ± 0.65	40.05cd ± 0.65	24.95b ± 0.044

OA: organic amendments, FYM: farmyard manure, PM: press mud. Values presented in the tables are the mean of four replicates with ±SE and different letters with each value indicating a significant difference at *p* < 0.05.

## Data Availability

Not applicable.
